# Genetic Diversity and Population Structure of Moroccan Isolates Belong to *Alternaria* spp. Causing Black Rot and Brown Spot in Citrus

**DOI:** 10.1155/2021/9976969

**Published:** 2021-11-23

**Authors:** Lamyaa Zelmat, Joseph Mbasani Mansi, Sarra Aouzal, Fatima Gaboun, Slimane Khayi, Mohammed Ibriz, Mohammed El Guilli, Rachid Mentag

**Affiliations:** ^1^Plant Pathology and Postharvest Quality laboratory, Plant Protection Research Unit, Regional Center of Agricultural Research of Kénitra, National Institue of Agricultural Research, El Menzeh Km 9, 14000, Kénitra, Morocco; ^2^Biotechnology Research Unit, Regional Center of Agricultural Research of Rabat, National Institute of Agricultural Research, Avenue Ennasr, BP 415 Rabat Principale, 10090, Rabat, Morocco; ^3^Department of Biology, Genetics and Biometrics Laboratory, Faculty of Sciences, Ibn Tofail University, Kénitra, Morocco; ^4^Department of Biology, Higher Institute of Medical Techniques (ISTM) Kinshasa, Democratic Republic of the Congo; ^5^Agro-Food and Health Laboratory, Faculty of Science and Techniques, Hassan First University of Settat, Settat, Morocco

## Abstract

*Alternaria alternata* is one of the most important fungi causing various diseases on citrus worldwide. In Morocco, Alternaria black rot (ABR) and Alternaria brown spot (ABS) are two major diseases causing serious losses in commercial cultivars of citrus. The aim of the present work was to study the genetic diversity and the population structure of isolates belonging to sect. *Alternaria* obtained from infected citrus fruits, collected from seven provinces at different locations in Morocco (markets, packinghouses, and orchards). Forty-five isolates were analyzed by sequence-related amplified polymorphism (SRAP) markers, and cluster analysis of DNA fragments was performed using UPGMA method and Jaccard coefficient. Cluster analysis revealed that isolates were classified in four distinct groups. AMOVA revealed also a large extent of variation within sect. *Alternaria* isolates (99%). The results demonstrate that no correlation was found among SRAP pattern, host, and geographical origin of these isolates. Population structure analyses showed that the *Alternaria* isolates from the same collection origin had almost a similar level of admixture.

## 1. Introduction

Citrus (*Citrus* spp., Rutaceae) is one of the most important crops due to its high annual worldwide production. A total of 124 million tons (Mt) was produced in 2016 [1]. Citrus are widely cultivated in over 140 countries around the world [2]. According to USDA [3], Morocco is one of three major producing countries of citrus in the Mediterranean region due to its favorable weather and increased acreage. In 2019, the Moroccan citrus cultivation area reached more than 129,000 ha generating a total production of 2.6 Mt [4]. Nonetheless, this crop is constantly threatened by fungal diseases that cause significant losses either in the field or after harvest leading to important economic impacts.

*Alternaria* species are considered a major threat to the citrus fruit industry worldwide causing black rot and brown spot diseases during degreening, storage, transportation, and marketing [5–7]. In 2015 and 2016, *Alternaria* spp. were the most prevalent pathogen causing the diseases of Mandarin fruit in California at 53.5% and 83.1%, respectively [5]. According to Akimitsu et al. [8], Alternaria brown spot (ABS) is a highly infectious disease of tangerines and their hybrids that occurs worldwide. It causes decrease in productivity and commercial value of fruit [9]. ABR is currently considered a serious postharvest problem, since the pathogen can occur abundantly in the central columella of the fruit without any manifestation of external symptoms and can be found in most citrus production areas [10].

Alternaria brown spot of tangerines (ABS) caused by the fungus *Alternaria alternata* (Fr.) Keissl [8, 11] is one of the four major *Alternaria* diseases affecting susceptible citrus genotypes around the world. The tangerine pathotype produces brown to black lesions on young leaves, twigs, and fruits with or without a chlorotic halo and causes corky lesions on mature fruit [8, 12]. *Alternaria* species cause three other diseases in citrus, including Alternaria leaf spot of rough lemon and rangpur lime [13], Alternaria black rot of many citrus cultivars [13], and Mancha foliar of Mexican lime [14]. The ABR pathogen causes black-colored infection developed in the central axis of fruit habitually without any symptoms on the rind surface [7]. The same symptoms have been observed on *Citrus sinensis* fruits, artificially inoculated with *Alternaria alternata* [15]. In addition, this pathogen can also produce stem end rot when the fungus pervades into the peel and juice sac of fruit before its harvest and continues to extend outwards later [10]. According to Peever et al. [16], there is a great morphological similarity among the citrus-associated isolates that have a small catenulate spores and that are considered to be distinct pathotypes of the same species, *Alternaria alternata*. The *Alternaria* species causing citrus brown spot were identified as belonging to *Alternaria alternata* and *A. arborescens* species complexes [17].

To gain insights about *Alternaria* spp. population circulating in Morocco, genetic diversity studies of the *Alternaria* pathogen are an important approach to understand its worldwide evolution and determine the population structure of this fungus. In this context, several profiling methods have been widely used to characterize the genetic variability of citrus-associated *Alternaria* species. These techniques include random amplified polymorphic DNA (RAPD) [18–20], amplified fragment length polymorphism (AFLP) [21, 22], intersimple sequence repeat (ISSR) [23], and restriction fragment length polymorphism **(**RFLP) [24]. In comparison with other molecular marker techniques, the sequence-related amplified polymorphism (SRAP) is relatively simple and very informative [25]. As demonstrated by [26], it is easier than AFLP markers and more reproducible than RAPD technique. Presently, SRAP markers were used to investigate genetic diversity of different fungi and have been successful for *Ganoderma lucidum* [27], *Sclerotinia sclerotiorum* [28], *Auricularia auricular* [29], *Lentinula edodes* [30], *Tricholoma matsutake* [31], *Auricularia auricula-judae* [32], *Rhizoctonia solani* [33], and *Pyricularia oryzae* [34].

Different phylogenetic loci were used to identify the species within *Alternaria* sect. *alternata* associated with citrus rots. In particular, the internal transcribed spacer (ITS) has been extensively used [11, 35–38], as well as Alternaria major allergen 1 (Alt a1) [15, 39], endopolygalacturonase (endo-PG) [7, 13, 40, 41], SCAR markers (OPA 1–3 and OPA 2–1) [40, 41], and the beta-tubulin gene (*β*-tub) [11, 20].

To date, there is no study reporting the genetic diversity of Moroccan *Alternaria* in citrus crop. Due to the abundance of *Alternaria* species in Morocco [42–44] which is being among the important exporters of citrus worldwide, our objectives for this study were (i) to analyze the genetic diversity and population structure of *Alternaria* spp. isolated from citrus fruit in Morocco using SRAP markers and (ii) to confirm the identity of citrus-associated isolates of *Alternaria* circulating in Morocco.

## 2. Materials and Methods

### 2.1. Fungal Isolates

**Figure 1:**
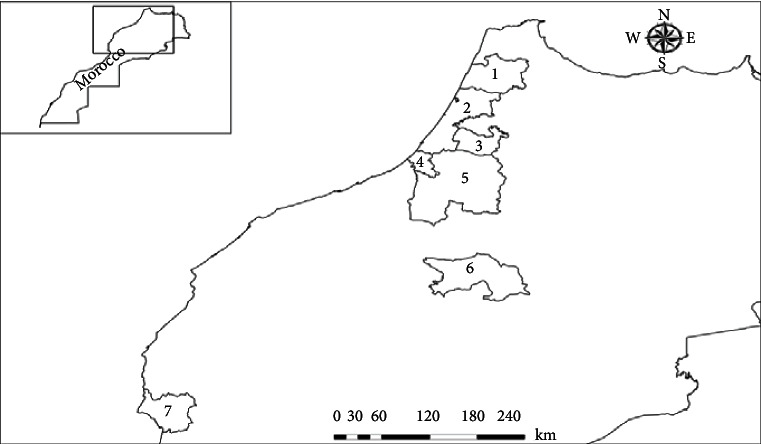
Geographic locations of the seven provinces where *Alternaria* sp. isolates were collected: 1—Larache, 2—Kénitra, 3—Sidi Slimane, 4—Salé, 5—Khémisset, 6—Beni Mellal, and 7—Agadir.

Forty-five isolates of *Alternaria* spp. were collected from seven provinces in Morocco from markets, packinghouses, and orchards during the period 2017-2018 (Table 1) (Figure 1). Infected fragments of 5 mm in diameter were sampled from each *Alternaria* symptomatic fruit, drenched in a 5% sodium hypochlorite solution for 2 min, and rinsed in sterilized distilled water. Then, all samples were placed into separate potato dextrose agar medium (PDA) and incubated at 25°C. The morphological identification of conidia was realized after 3 or 4 days of incubation using a microscope at an objective of 40x. *Alternaria* sp. colonies were transferred to fresh PDA medium and stored at 4°C until use.

### 2.2. DNA Extraction

For DNA extraction, mycelia were collected in single-spore cultures of the isolates grown in PDA for 7 days at 25°C. The total DNA was extracted following the modified CTAB method [45]. A total of 50 mg of mycelia was mixed with 1 ml of CTAB extraction buffer (0.15 M Tris-HCl, 1.05 M NaCl, 0.03 M EDTA, 2% (*w*/*v*) CTAB, and 0.02% (*v*/*v*) of *β*-mercaptoethanol) and incubated to 65°C for 1 hour. A volume of 0.6 ml of chloroform isoamyl : alcohol (24 : 1—*v*/*v*) was added, and the mixture was centrifuged at 13000 rpm for 10 min. The supernatant was recovered in a new tube, and DNA precipitated overnight by isopropanol. Finally, the DNA was washed with 200 *μ*l of ethanol (75%) and dissolved with sterile ultrapure water. The purity and quality of extracted DNA were assessed by a spectrophotometer at 230, 260, and 280 nm. For SRAP reactions, the concentration was adjusted to 25 ng/*μ*l.

### 2.3. SRAP Analysis and Polymerase Chain Reaction

SRAP-PCR analysis was performed by assessing for polymorphism, 30 combinations of primers (five forward/six reverse) (Table 2), following the protocol described by [26]. The best primer combinations that generated clear polymorphism with reproducible PCR products were used on the 45 DNA samples. All SRAP reactions were prepared at a final volume of 10 *μ*l containing 1 U of Taq DNA polymerase, 1X buffer, 0.5 *μ*M of each primer, and 25 ng of DNA template. The amplification of DNA was programmed with an initial denaturation at 94°C for 5 min followed by 5 cycles with denaturation, annealing, and extension, respectively, at 94°C, 35°C, and 72°C for 1 min for each step. Then, 35 cycles were added with the annealing temperature set at 50°C. The PCR products were analyzed on polyacrylamide gel stained by ethidium bromide and visualized using UV transilluminator.

### 2.4. Molecular Phylogeny

To ascertain the identity of the fungal isolates, 11 representative *Alternaria* strains (Elm1, A3, K5, G5, E3, I2, Sds6, La1, Sd2, Ag21, and, Sds8) were selected from our collection for molecular identification. Internal transcribed spacer (ITS) region of rDNA was amplified. PCR products were Sanger sequenced (Secugen, Madrid, Spain). To perform the phylogenetic analysis, ITS sequences of reference strains were retrieved from GenBank and used (Table 3). Multiple sequence alignment of the ITS sequences was conducted using ClustalW, and the phylogenetic tree was inferred using the neighbour-joining method [46] with 1000 bootstrap replications. Analyses were conducted in MEGA version X [47].

### 2.5. Statistical Analyses

The molecular data of variability were scored for the presence or absence of the amplification profile for each primer combination, respectively, as 1 or 0. Efficiency of SRAP markers was evaluated with the polymorphism rates of primers and polymorphism information content (PIC). The PIC was calculated as 2*ƒ*_*i*_ × (1 − *ƒ*_*i*_), where *f*_*i*_ is the frequency of band presence for locus *I* [48].

The percentage of polymorphic loci, heterozygosity, and Shannon's information index were calculated for the genetic diversity analysis using GenAlEX version 6.5 [49]. Molecular variance analysis (AMOVA) was performed to determine the genetic variance within and among isolates according to their geographic origins [50]. Based on the Jaccard dissimilarity coefficient, the dendrogram was constructed using DARwin version 6 [51] and employing the unweighted pair group method with an arithmetic average (UPGMA) cluster analysis.

STRUCTURE software v.2.3.4 [52] and Bayesian distinct Monte Carlo Markov Chain (MCMC) algorithm were used in this study to identify the population structure of *Alternaria* spp. isolates from citrus fruits. Each simulation considered in 10,000 iterations after a length burning period of 1,000. The true number of clusters (*K*) of the admixture test was estimated, afterward, from the Structure Harvester web tool [53].

## 3. Results

### 3.1. SRAP Analysis

A total of 30 primer combinations were initially tested for SRAP analysis. The result allowed to select six primer combinations showing the highest polymorphism (ME2/EM6, ME4/EM6 ME5/EM2, ME5/EM6, ME2/EM3, and ME3/EM6). The total number of bands generated by each primer varied from 10 (ME3/EM6) to 32 (ME2/EM6) with an average of 22.3. The polymorphism rate ranged from 99.3 to 100%, with an average of 99% of number of polymorphic bands (Table 4). The PIC values varied from 0.22 (ME3/EM6) to 0.35 (ME5/EM2).

### 3.2. Genetic Diversity

According to SRAP marker analysis, the results revealed various levels of genetic diversity indices (Table 5). The percentage of polymorphic loci within populations varied from 15.67 to 94.03%, in Larache and Kénitra populations, respectively. The highest values of Shannon's information index (*I*), expected heterozygosity (He), number of different alleles (Na), and number of effective alleles (Ne) were observed in Kénitra, with 0.41, 0.26, 1.89, and 1.44, respectively, while the lowest values were observed in Larache with 0.09, 0.06, 0.62, and 1.11, respectively.

### 3.3. Genetic Distance

The genetic distance was calculated to determine the similarity among the isolates based on their geographical origin. The results revealed that the genetic distance was very low between Kénitra and Sidi Slimane populations (0.030), while it was very high between Salé and Khemisset, Salé and Sidi Slimane, then between Larache and Beni Mellal (0.212) (Table 6). Molecular variance analysis (AMOVA) was performed to estimate genetic differentiation among populations and within populations (PhiPT). Accordingly, AMOVA test showed the highest genetic variation within populations (99%), whereas the level of interpopulation variation was only 1% (Table 7).

### 3.4. Cluster Analysis

**Figure 2:**
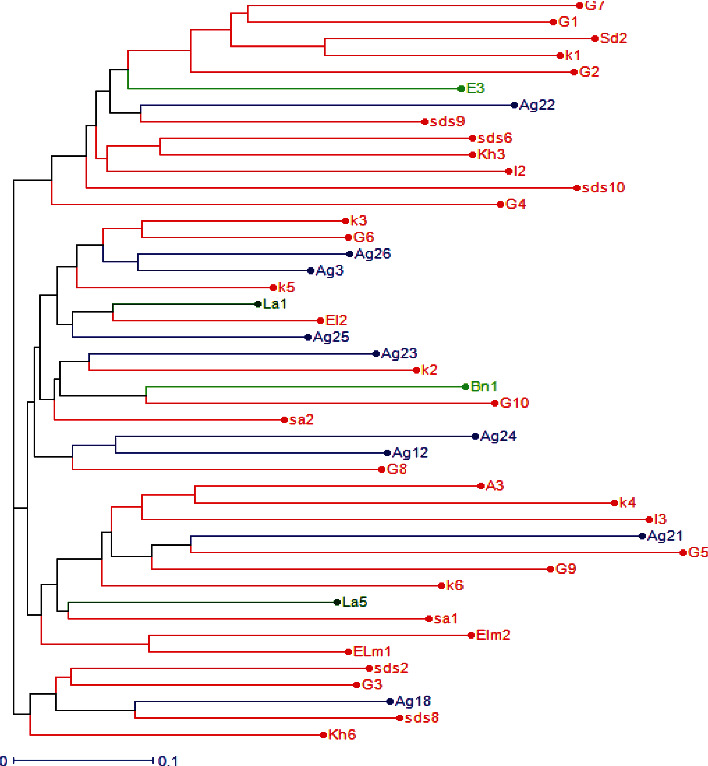
Dendrogram of 45 *Alternaria* isolates from Citrus collected from seven provinces in Morocco. Unweighted pair group method with arithmetic mean (UPGMA) cluster analysis was based on Jaccard's coefficient. A3, Elm1, Elm2, K1, K2, K3, K4, K6, K5, Kh6, Sa2, Sd2, Sds2, Sds9, Sds10, Sds8, Sds6, Ag3, Ag12, Ag18, Ag22, Ag23, Ag24, Ag25, Ag26, Ag21, and Bn1—oranges; El2, La5, and La1— Mandarins; G1, G2, G3, G4, G6, G7, G8, G9, G10, G5, I2, I3, Kh3, and Sa1—lemons; E3—clementines.

UPGMA cluster analysis of SRAP data, using Jaccard coefficient, revealed four distinct groups at 60% dissimilarity mean level, with a maximum of 86% and a minimum of 25%. The dendrogram illustrated in Figure 2 showed that the first group included 5 isolates originating from Khémisset, Sidi Slimane, Agadir, and Kénitra. The second group accommodated 11 isolates obtained from Khémisset, Agadir, Kénitra, Salé, and Larache. The third contained 16 isolates and obtained from Agadir, Kénitra, Salé, Larache, and Beni Mellal. The last group included 13 isolates collected from Khémisset, Sidi Slimane, Agadir, Kénitra, and Beni Mellal. Considering the genetic variability between the 45 isolates studied, no correlation was shown with their geographic location or their host plant.

### 3.5. Population Structure

**Figure 3:**
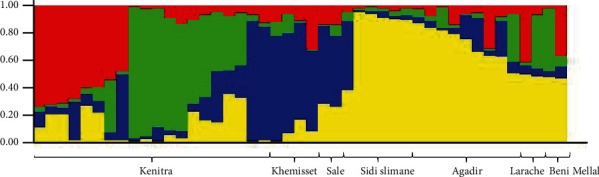
Genetic assignment of the *Alternaria* populations using the STRUCTURE program at *K* = 3 and Bayesian method.

The *Alternaria* isolates were genetically structured using the STRUCTURE software. The true number of clusters (*K*) was identified based on the Bayesian approach. Structure Harvester web tool findings indicated that the highest value of delta *K* equals 3. The Bayesian cluster analysis revealed, afterward, four main groups with the best identified *K* value (Figure 3). Each colored column represented an individual isolate of *Alternaria* population. The isolates from the same collection origin had almost a similar level of admixture and were assigned in the same genetic group. However, Kénitra individuals were classified in two different groups, while the most isolates from Khémisset and Salé were assigned in the third group. The isolates from Sidi Slimane, Agadir, Larache, and Beni Mellal provinces were assigned in the last.

### 3.6. Molecular Phylogeny

**Figure 4:**
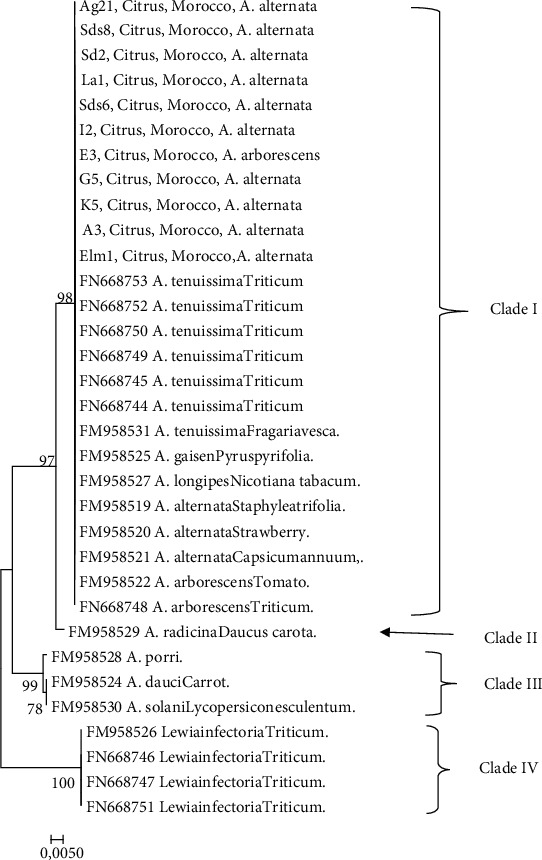
Phylogenetic tree based on the internal transcribed spacer (ITS) sequences of 11 isolates of *Alternaria* spp. obtained from citrus fruit. The bootstrap value support is indicated in the branches.

Phylogenetic tree generated using ITS sequences highlighted four distinct clades with high bootstrap value support (1000) (Figure 4). The topology positioned the 11 *Alternaria* isolates from citrus within Clade I with five other references strains for *Alternaria* isolates obtained from citrus with five other reference strains for *Alternaria alternata*, *A. tenuissima*, *A. arborescens*, *A. gaisen*, and *A. longipes*. Clade II includes the only *A. radicina* strain. *Alternaria porri*, *A. dauci*, and *A. solani* formed Clade III, while *Lewia infectoria* constitute the last Clade.

## 4. Discussion

Sequence-related amplified polymorphism (SRAP) is an adequate molecular marker widely used to assess the genetic diversity in plants and fungi [32, 34, 54–56]. Compared to other molecular markers, SRAP is the simplest and the most reproducible technique of genome-wide fragments and remains an inexpensive and highly variable method [57]. It is generally used to construct the genetic map and analysis of genetic diversity and to compare genomes [58]. Many recent studies have used the SRAP technique to analyze the genetic diversity of different fungal pathogens. Tripathi and Dubey [33] studied the genetic diversity of 89 isolates of *Rhizoctonia solani* infecting pulse crops in India, using this molecular marker. Longya et al.[34] used both SRAP and ISSR markers to examine the genetic variation of 59 rice blast fungus strains, *Pyricularia oryzae*, in Thailand.

In the present study, SRAP marker was used to evaluate the level of genetic diversity within and among 45 isolates of *Alternaria* spp. obtained from Moroccan citrus. The results indicates a total of 133 of polymorphic bands generated by six primer combinations with an average of 22.16 bands per primer combination. The mean polymorphism rate (99%) and Shannon's index (0.27) confirmed also high levels of genetic diversity of the isolates. The same pattern was reported by [18], who observed high genetic diversity among 45 isolates of *A. alternata* from citrus in southern Iran, based on RAPD markers using eight primers. The average of amplified bands was 27.12. Furthermore, the Brazilian isolates of *A. alternata* collected from Tangerine were characterized based on ISSR markers and 20 polymorphic bands, the isolates revealing low genetic variability in three groups and high diversity only in last group [23].

Several research used the Polymorphism Information Content Values (PICv) to assess the genetic diversity among species. According to [59], the high, medium, or low locus polymorphism is compatible, respectively, with, PIC > 0.5, 0.5 > PIC > 0.25, and PIC < 0.25. In present study, the PICv ranged from 0.22 to 0.35 with an average value of 0.28. Six values of PIC exceeded 0.25, and only one was less than 0.25. These results showed that the SRAP markers are an efficient tool to determine the level of genetic diversity of *Alternaria* isolates.

The dendrogram constructed in this study using the UPGMA method distinguished four main groups, based on the molecular diversity of isolates and their geographic origins. The clustering results showed no significant correlation between the genetic variability of *Alternaria* isolates and their distribution in Morocco. These results are in accordance with [22] who assessed the genetic diversity of *A. alternata* populations sampled from tangerines in Italy and reported that there was no correlation between geographical origin, host, and year of isolation. In another study, a slight effect of host plant and geographical location on genetic variation in *A. alternata* obtained from seeds of *Amaranthus* was observed using the RAPD technique [60]. As described by [61], the absence of relationship among the origin and molecular diversity of this collection was likely attributable to the great mobility and the uniform dispersal by wind of *A. alternata* spores, which can cover enormous distances. In contrast, other works reported that the genetic diversity of *A. alternata* collected from Citrus [19] and potatoes [62] was dependent on their sampled locations, hosts, or year of isolation. Additionally, [63] detected a high level of molecular diversity among *A. solani* originating from the same field of potatoes in Germany.

Our findings of AMOVA test showed the highest genetic variation within populations (99%), whereas the level of interpopulation variation was only 1%. This is in accordance with [64] who observed that the variation within Australian populations of *A. brassicicola* (86%) was higher than among them (14%). In another study, [65] found high genetic variation within *A. alternata* from *Pinus tabulaeformis* detected by random amplified microsatellites (RAMS). The results showed that there was no correlation between the fungal genotypes and host tissue. Furthermore, [66] detect high genetic diversity within populations of *A. solani* in China using SSR markers.

The phylogeny analysis results showed that all the 11 *Alternaria* isolates of the present study selected were assembled with five other strains of *Alternaria* used as references. In particular, the ITS sequences of *Alternaria* species isolated from citrus were identical to those of *A. alternata*, *A. tenuissima*, *A. arborescens*, *A. gaisen*, and *A. longipes*. This result is in accordance with the finding in [16], which reported a similarity among nine *Alternaria* isolates from citrus and seven other species including *A. alternata*, *A. longipes*, *A. infectoria*, *A. tenuissima*, *A. gaisen*, *A. arborescens*, and *A. mali* based on MtLSU phylogenies. In another study, Armitage et al. [39] based on sequencing of five phylogenetic loci (endo-PG, Alt a1, TMA22, PGS1, and REV3) and morphological analysis reported that the citrus-associated strains of *Alternaria* studied were grouped with *A. alternata*, *A. tenuissima*, and *A. mali* (EGS references). Their findings indicated that *A. alternata* subsp. *arborescens*, *A. alternata* subsp*. gaisen*, and *A. alternata* subsp. *tenuissima* were considered subspecies within the *A. alternata* species group. In addition, among twenty isolates of *Alternaria* spp. collected from citrus, eighteen were referable to *A. alternata* CBS references and two referable to *A. arborescens* CBS references based on the ITS and endo-PG sequencing [11]. These authors found a higher variability within *A. alternata* species when the phylogenetic analysis is based on endo-PG, ITS, *β*-tub, and SCAR marker (OPA1-3 and OPA2-1) sequences.

## 5. Conclusion

The present study concerned the assessment of genetic differentiation of *Alternaria* isolates originating from seven provinces in Morocco. SRAP markers revealed high polymorphism rate with an average of 99%. Furthermore, AMOVA results demonstrate that the level of genetic diversity was higher within the Moroccan *Alternaria* populations (99%) than among them (1%). Cluster analysis constructed according to the genetic diversity of the isolates and their distribution generated four distinct groups. These findings indicate that SRAP markers could be an adequate method to be used for genetic structure and molecular differentiation studies among and within *Alternaria* populations. Molecular identification by sequencing of the ITS region confirmed that the selected Moroccan strains belong to the *A. alternata* species complex. The reported knowledge of genetic diversity and population structure in this study furthers our understanding of the *Alternaria* pathogens on citrus. This provides importance for selecting the resistance citrus cultivars and reduces, furthermore, the pre- and postharvest losses caused by species of *Alternaria* sect. *alternata*. The collection from other provinces in Morocco could be appropriate to evaluate the variability of *Alternaria* on a large scale.

## Figures and Tables

**Table 1:** Isolates of *Alternaria* spp. obtained from citrus fruits during 2017-2018.

Isolate	Host	Date	Province/site	Location (GPS)	Accession number
A3	Oranges	December, 2017	Kénitra/orchard	N 34° 30′ 52,964^″^ W 6° 18′ 3,789^″^	MW575592
Elm1	Oranges	March. 2018	Kénitra/orchard	N 34° 17′ 38,99^″^ W 6° 29′ 3,97^″^	MW616575
Elm2	Oranges	March. 2018	Kénitra/orchard	N 34° 17′ 38,99^″^ W 6° 29′ 3,97^″^	—
K1, K2, K3, K4, K6	Oranges	March, 2018	Kénitra/market	N 34° 14′ 59,18^″^ W 6° 32′ 58,14^″^	—
K5	Oranges	March, 2018	Kénitra/market	N 34° 14′ 59,18^″^ W 6° 32′ 58,14^″^	MW616574
El2	Mandarins	February, 2018	Kénitra/orchard	N 34° 17′ 38,99^″^ W 6° 29′ 3,97^″^	—
G1, G2, G3, G4, G6, G7, G8, G9, G10	Lemons	March, 2018	Kénitra/market	N 34° 15′ 16,315^″^ W 6° 36° 58,09^″^	—
G5	Lemons	March, 2018	Kénitra/market	N 34° 15′ 16,315^″^ W 6° 36° 58,09^″^	MW616570
I2	Lemons	January, 2018	Kénitra/market	N 33° 46′ 43,42^″^ W 6° 7′ 23,34^″^	MW616569
I3	Lemons	January, 2018	Kénitra/market	N 33° 46′ 43,42^″^ W 6° 7′ 23,34^″^	—
Kh3	Lemons	April, 2018	Khémisset/market	N 33° 49′ 31,824^″^ W 6° 4′ 20,303^″^	—
Kh6	Oranges	April, 2018	Khémisset/market	N 33° 49′ 31,824^″^ W 6° 4′ 20,303′	—
Sa1	Lemons	April, 2018	Salé/market	N 34° 2′ 56,81^″^ W 6° 47′ 23,01^″^	—
Sa2	Oranges	April, 2018	Salé/market	N 34° 2′ 56,81^″^ W 6° 47′ 23,01^″^	—
Sd2	Oranges	March, 2018	Sidi Slimane/market	N 34° 16′ 1,476^″^ W 5° 55′ 30,732^″^	MW616571
Sds2, Sds9, Sds10	Oranges	April, 2018	Sidi Slimane/market	N 34° 16′ 1,476^″^ W 5° 55′ 30,732^″^	
Sds8	Oranges	April, 2018	Sidi Slimane/market	N 34° 16′ 1,476^″^ W 5° 55′ 30,732^″^	MW616567
Sds6	Oranges	April, 2018	Sidi Slimane/market	N 34° 16′ 1,476^″^ W 5° 55′ 30,732^″^	MW616573
La5	Mandarins	January, 2018	Larache/packinghouse	N 35° 0′ 7,08^″^ W 6° 11′ 58,23^″^	—
La1	Mandarins	January, 2018	Larache/packinghouse	N 35° 0′ 7,08^″^ W 6° 11′ 58,23^″^	MW616572
Ag3, Ag12	Oranges	May, 2018	Agadir/market	N 30° 24′ 44,37^″^ W 9° 34′ 48,63^″^	—
Ag18, Ag22, Ag23, Ag24, Ag25, Ag26	Oranges	July, 2018	Agadir/market	N 30° 24′ 44,37^″^ W 9° 34′ 48,63^″^	—
Ag21	Oranges	July, 2018	Agadir/market	N 30° 24′ 44,37^″^ W 9° 34′ 48,63^″^	MW616576
Bn1	Oranges	April, 2018	Beni Mellal/market	N 32° 20′ 40,469^″^ W 6° 20′ 45,653^″^	—
E3	Clementines	December, 2017	Beni Mellal/packinghouse	N 32° 20′ 40,469^″^ W 6° 20′ 45,653^″^	MW616568

**Table 2:** Forward and reverse sequences of SRAP primers used in this study.

Forward primer names	Sequences	Reverse primer names	Sequences
ME1	5′-TGAGTCCAAACCGGATA-3′	EM1	5′-GACTGCGTACGAATTAAT-3′
ME2	5′-GAGTCCAAACCGGAGC-3	EM2	5′-GACTGCGTACGAATTTGC-3
ME3	5′-TGAGTCCAAACCGGAAT-3′	EM3	5′-GACTGCGTACGAATTGAC-3′
ME4	5′-TGAGTCCAAACCGGACC-3′	EM4	5′-GACTGCGTACGAATTTGA-3′
ME5	5′-TGAGTCCAAACCGGAAG-3′	EM5	5′-GACTGCGTACGAATTAAC-3′
		EM6	5′-GACTGCGTACGAATTGCA-3′

**Table 3:** Reference strains of *Alternaria* spp. used in this study and their accession numbers.

Species	Strain	Host/origin	GenBank accession number (ITS)
*A. arborescens*	CBS 109730	Tomato	FM958522
	MAP05a	*Triticum*, grain	FN668748
*A. alternata*	CBS 117130	Potted strawberry tree	FM958521
	CBS 117143	*Capsicum annuum*, fruit	FM958520
	CBS 154.31	*Staphylea trifolia*	FM958519
*A. tenuissima*	MAP02	*Triticum*, grain	FN668745
	MAP09	*Triticum*, grain	FN668752
	MAP01	*Triticum*, grain	FN668744
	MAP07	*Triticum*, grain	FN668750
	MAP10	*Triticum*, grain	FN668753
	MAP06	*Triticum*, grain	FN668749
	CBS 880.95	*Fragaria vesca*	FM958531
*A. dauci*	CBS 101592	Carrot seeds	FM958524
*A. solani*	CBS 347.79	Tomato, fruit	FM958530
*A. gaisen*	CBS 632.93	*Pyrus pyrifolia*, leaf	FM958525
*A. longipes*	CBS 917.96	*Nicotiana tabacum*	FM958527
*A. porri*	CBS 109.41	—	FM958528
*A. radicina* var. *radicina*	CBS 245.67	*Daucus carota*	FM958529

**Table 4:** Analysis of SRAP primer combinations results.

SRAP primers	Total number of bands	Number of polymorphic bands	Polymorphism rate (%)	PIC values
ME2/EM6	32	32	100	0.30
ME4/EM6	15	14	93,33	0.26
ME5/EM2	26	26	100	0.35
ME5/EM6	26	26	100	0.27
ME2/EM3	25	25	100	0.29
ME3/EM6	10	10	100	0.22
Total	134	133	—	—
Mean	22.3	22.2	99	0.28

**Table 5:** Summary of genetic diversity indices of *Alternaria* isolates collected from seven provinces of Morocco based on SRAP marker.

Pop	*P* (%)	Na	Ne	*I*	He
Kénitra	94.03	1.896	1.441	0.410	0.266
Khémisset	61.94	1.313	1.370	0.330	0.219
Salé	25.37	0.806	1.179	0.153	0.105
Sidi Slimane	70.90	1.455	1.363	0.339	0.220
Agadir	71.64	1.455	1.435	0.370	0.248
Larache	15.67	0.627	1.111	0.095	0.065
Beni Mellal	33.58	0.873	1.237	0.203	0.139
Mean	53.30	1.204	1.305	0.271	0.180

P (%): percentage of polymorphic loci; Na: number of different alleles; Ne: number of effective alleles; *I*: Shannon's information index; He: expected heterozygosity.

**Table 6:** Pairwise population matrix of Nei genetic distance.

	Kénitra	Khémisset	Salé	Sidi Slimane	Agadir	Larache	Beni Mellal
Kénitra	0.000						
Khémisset	0.042	0.000					
Salé	0.159	0.212	0.000				
Sidi Slimane	0.030	0.041	0.212	0.000			
Agadir	0.033	0.060	0.158	0.041	0.000		
Larache	0.132	0.162	0.153	0.169	0.127	0.000	
Beni Mellal	0.109	0.106	0.210	0.092	0.114	0.212	0.000

**Table 7:** Analyses of molecular variance (AMOVA) for *Alternaria* isolates by SRAP markers.

Source	Df	SS	MS	Est. var.	%	PhiPT	*P*
Among populations	6	128,147	21,358	0.175	1%	0.009	0.414
Within populations	38	774,431	20,380	20,380	99%		
Total	44	902,578		20,555	100%		

Df: degree of freedom; SS: sums of squares; MS: mean squares; Est. var: estimate of variance; %: percentage of total variation; PhiPT: phi-statistics probability level after 999 permutations; *P* is based on 999 permutations.

## Data Availability

Accessions Nos. MW575592, MW616575, MW616574, MW616570, MW616569, MW616571, MW616567, MW616573, MW616572, MW616576, and MW616568 are already available in NCBI.
